# Gluconeogenesis, lipogenesis, and HBV replication are commonly regulated by PGC-1α-dependent pathway

**DOI:** 10.18632/oncotarget.3050

**Published:** 2015-02-03

**Authors:** Hong-Jhih Jhuang, Wei-Hsiang Hsu, Kuan-Ting Lin, Shih-Lan Hsu, Feng-Sheng Wang, Chen-Kung Chou, Kuen-Haur Lee, Ann-Ping Tsou, Jin-Mei Lai, Sheau-Farn Yeh, Chi-Ying F. Huang

**Affiliations:** ^1^ Institute of Biochemistry and Molecular Biology, National Yang-Ming University, Taipei, Taiwan; ^2^ Institute of Biopharmaceutical Sciences, National Yang-Ming University, Taipei, Taiwan; ^3^ Institute of Biomedical Informatics, National Yang-Ming University, Taipei, Taiwan; ^4^ Department of Medical Research, Taichung Veterans General Hospital, Taichung, Taiwan; ^5^ Department of Chemical Engineering, National Chung Cheng University, Chiayi, Taiwan; ^6^ Department of Biomedical Sciences, Chang Gung University, Taoyuan, Taiwan; ^7^ Department of Life Science, Fu-Jen Catholic University, New Taipei City, Taiwan; ^8^ Graduate Institute of Cancer Biology and Drug Discovery, College of Medical Science and Technology, Taipei Medical University, Taipei, Taiwan; ^9^ Department of Biotechnology and Laboratory Science in Medicine, National Yang-Ming University, Taipei, Taiwan

**Keywords:** *Graptopetalum paraguayense*, HBV, Gluconeogenesis, Lipogenesis, PGC-1α

## Abstract

PGC-1α, a major metabolic regulator of gluconeogenesis and lipogenesis, is strongly induced to coactivate Hepatitis B virus (HBV) gene expression in the liver of fasting mice. We found that 8-Br-cAMP and glucocorticoids synergistically induce PGC-1α and its downstream targets, including PEPCK and G6Pase. Also, HBV core promoter activity was synergistically enhanced by 8-Br-cAMP and glucocorticoids. *Graptopetalum paraguayense* (GP), a herbal medicine, is commonly used in Taiwan to treat liver disorders. Partially purified fraction of GP (named HH-F3) suppressed 8-Br-cAMP/glucocorticoid-induced G6Pase, PEPCK and PGC-1α expression and suppressed HBV core promoter activity. HH-F3 blocked HBV core promoter activity via inhibition of PGC-1α expression. Ectopically expressed PGC-1α rescued HH-F3-inhibited HBV surface antigen expression, HBV mRNA production, core protein levels, and HBV replication. HH-F3 also inhibited fatty acid synthase (FASN) expression and decreased lipid accumulation by down-regulating PGC-1α. Thus, HH-F3 can inhibit HBV replication, gluconeogenesis and lipogenesis by down-regulating PGC-1α. Our study indicates that targeting PGC-1α may be a therapeutic strategy for treatment of HBV infections. HH-F3 may have potential use for the treatment of chronic hepatitis B patients with associated metabolic syndrome.

## INTRODUCTION

Hepatitis B virus (HBV) infection frequently results in acute and chronic hepatitis, which could lead to liver cirrhosis and hepatocellular carcinoma (HCC). Approximately 350 million people worldwide are infected by HBV [1]. Based on recent studies, the impact of non-alcoholic fatty liver disease (NAFLD) or non-alcoholic fatty steatohepatitis as the hepatic manifestation of diabetes/obesity has been clearly linked to liver disease progression (fibrosis, cirrhosis) and HCC risk in several clinical or molecular studies. The risk of HCC increases more than 100-fold in HBV carriers with both obesity and diabetes, indicating the synergistic effects of metabolic factors and hepatitis [2, 3]. Four proteins originate from the HBV genome, including polymerase, surface antigen, core, and HBx proteins. HBx and core proteins are associated with HBV-related pathogenesis [4, 5]. The X gene encodes the X protein (HBx), which has transactivating properties and might be important in hepatic carcinogenesis. The core gene encodes the core nucleocapsid protein (important in viral packaging) [6]. *In vitro* studies suggest that core promoter mutations increase HBV replication [7].

Diminishing viral replication remains crucial for patients because it not only prevents further infection but also attenuates the inflammation response to viral expression. Currently, no therapeutic strategy that could completely eradicate HBV from the host is hitherto available. The current anti-HBV drugs of choice are members of the nucleoside analog family, including lamivudine [3TC], adefovir, andentecavir. Because these drugs mainly target the viral polymerase [8], resistance and cross-resistance against nucleoside analogs have emerged after only one to two years of treatment [9]. The point mutations that lead to the emergence of resistance have also been identified recently [10]. Since viral replication elements have been targeted to stall HBV production, increasing attention is being focused on identifying anti-HBV agents unaffected by resistance. Therefore, the development of a new generation of anti-HBV agents with new modes of action is urgently needed.

Peroxisome proliferator-activated receptor-γ coactivator 1 (PGC-1α) plays a crucial role in the maintenance of glucose, lipid, and energy homeostasis in the liver. The elevated expression of PGC-1α may alter the metabolic properties of tissues and lead to various diseases with an underlying dysregulation of metabolism, such as obesity [11], diabetes [12], neurodegeneration [13], and cardiomyopathy [14]. Several reports have suggested that HBV adopts a mode of regulation similar to major gluconeogenesis genes in the liver, such as *PEPCK* and *G6Pase*, which are co-regulated by PGC-1α, HNF4α and FOXO1 [15, 16]. Interestingly, PGC-1α induces oxidative phosphorylation, and the expression of tricarboxylic acid cycle genes—such as *SLC25A1* and *ACLY*—also increases the expression of the *de novo* fatty acid synthesis enzymes, acetyl CoA carboxylase (*ACC*) and fatty acid synthase (*FASN*) [17]. The genes involved in the biosynthesis of lipids, such as FASN and SREBP-2, are up-regulated in HBV-transgenic mouse liver [18]. These findings imply that aberrations of lipid metabolism are also associated with chronic HBV infection.

*Graptopetalum paraguayense* (GP), a herbal medicine commonly used in Taiwan, is considered to have potentially beneficial effects by alleviating hepatic disorders, lowering blood pressure, relieving pain and infections, and inhibiting inflammation [19, 20]. In addition, GP extracts show a hepatoprotective effect via promoting antioxidative and anti-inflammatory properties against CCl_4_-induced oxidative liver damage [21]. Microarray profiling showed that the expression of most metabolism- and cell growth- and/or maintenance-related genes was recovered to near normal levels following GP treatment in a DMN-induced liver fibrosis model [22]. Moreover, the administration of GP ameliorated chemical-induced hepatic damage and fibrosis *in vivo* and suppressed hepatic stellate cell (HSC) and Kupffer cell activation *in vitro*, suggesting that GP most likely is a therapeutic drug for hepatic inflammation and fibrosis [23]. Furthermore, GP could improve carboxymethyllysine (CML)-induced hyperglycemia and results in a significant reduction in blood pressure, blood glucose, and lipid profiles in patients with metabolic syndrome after supplementation with water extracts of GP [24, 25]. The literature indicates a significant reduction in the blood glucose level and lipid profiles in subjects with metabolic syndrome after supplementation with water extracts of GP.

In this study, we found that GP and its partially purified fraction (HH-F3) could down-regulate PGC-1α expression, resulting in the inhibition of gluconeogenesis, lipogenesis, and HBV replication. These observations suggest that GP/HH-F3 is a potential agent to be used for the treatment of HBV-related fibrosis/cancer with metabolic syndrome.

## MATERIALS AND METHODS

### Reagents

Hepatitis B surface antigen (HBsAg) enzyme immunoassay (EIA) kits were purchased from GBC (General Biologicals, Taiwan). Dulbecco's modified Eagle's medium (DMEM) and fetal calf serum were obtained from Invitrogen Life Science Inc (Grand Island, NY). MTT (3-[4, 5-dimethylthiazol-2-yl]-2, 5-diphenyl-tetrazolium bromide), 8-bromoadenosine 3′, 5′-monophosphate sodium, bovine insulin, and Rapamycin were purchased from Sigma Chemical Co. (St. Louis, MO, USA).

### Cell culture

The human hepatoma Huh7 cell line was obtained from Dr. Zhong-Zhe Lin, National Taiwan University Hospital, Taiwan. The HepG2 cell line was purchased from Bioresource Collection and Research Center (BCRC, http://www.bcrc.firdi.org.tw/), Taiwan. The Mahlavu cell line was provided by Dr. Muh-Hwa Yang, Institute of Clinical Medicine, National Yang-Ming University, Taiwan. The Hep3B/T2 cell line can continuously secrete HBsAg into the culture medium [26]. The 1.3ES2 cell line is a clonal derivative of HepG2 cells in which the 1.3 copies of the entire HBV genome were stably integrated into the host genome [27]. Cultures of human hepatoma cells Huh7, HepG2, Mahlavu, Hep3B/T2 and 1.3ES2 were maintained in DMEM supplemented with 10% fetal calf serum and antibiotics (100 IU/ml each of penicillin and streptomycin) in a humidified atmosphere containing 5% CO_2_ and 95% air at 37°C. The cultures were passaged by trypsinization every 3–4 days.

### GP extraction and purification

The leaves of *Graptopetalum paraguayense* were ground and lyophilized into powder at −20°C and stored in a moisture buster at 25°C before extraction. First, 1.5 g of *Graptopetalum paraguayense* powder was vortexed with 10 ml of 100% methanol (MeOH) for 5 minutes and then centrifuged for 5 min. After removal of the supernatant, 10 mL of 30% DMSO was added to each pellet to resuspend them. The suspension was mixed by vortexing for 5 min, centrifuged twice for 5 min, and filtered using a 0.45-μm filter at room temperature. The 30% DMSO supernatant was either stored at −20°C as a 150-mg/ml stock solution (referred to as GP extracts) or fractionated into four fractions (F1–F4) by a Sephadex LH-20 column. Using the analysis of AURKA, AURKB, and FLJ10540 protein levels via Western blotting, active molecules were obtained in fraction 3, referred to as the HH-F3 fraction. The HH-F3 fraction was further analyzed by HPLC with a UV detector (Shimadzu SPD-M10A), a normal-phase HPLC column (Phenomenex Luna 5 μm Silica (2) 100 Å, 4.6 × 250 mm), and ^1^H- and ^13^C-NMR spectra to identify the structure of the active molecules. HH-F3 was then subjected to dialysis against water using a dialysis membrane (MWCO 12–14,000) (Spectrum Laboratories, Rancho Dominguez, CA) to obtain active compounds.

### 8-Bromo-cAMP/dexamethasone induction

Cultured human hepatoma Hep3B/T2 cells were treated with 0.5 mM 8-Bromo-cAMP (8-Br-cAMP) alone or 0.5 μM dexamethasone (Dex) alone or both for 30 min in serum-free DMEM medium, and then the different concentrations of HH-F3 were added in the serum-free DMEM for 24 h.

### Transient transfection and luciferase assay

Hep3B/T2 cells were transfected with pCP-Luc and pG6Pase-Luc (0.45 μg/mL) plasmid using Maestrofectin transfection reagent (Maestrogen, Taiwan). The transfected cells were changed to serum-free DMEM with drug for 24 h. To prepare total cell lysates from transfected cells for luciferase activity measurement, the medium was aspirated from the cell culture, and the cells were gently rinsed with PBS. Cells were scrapped from the plates using lysis buffer (0.1 M potassium buffer, 1% Triton X-100, 1 mM DTT, 2 mM EDTA) in 4°C for 15 min. Lysates were analyzed for luciferase activity using a luminometer and the Promega Luciferase Assay System as described by the manufacturer. Luciferase activities were normalized to the amount of protein in each lysate. For all transient transfections with the promoter-luciferase reporter construct, the luciferase activity level without drug treatment was set to one. The transfection efficiency was normalized using the activity of β-galactosidase as an internal control. The values are represented as the mean ± SD from at least three independent experiments.

### Plasmid construction

The HBV sequence used in this study is of the ayw subtype [28] and is derived from the GenBank accession number V01460 with the EcoRI site as nucleotide 1. To generate pXP-Luc, the XbaI-HindIII fragments containing the X promoter from pXP-CAT were inserted into the MluI-HindIII site of the pGL3-Basic vector (Promega). The pCP-Luc, pCPD1-Luc, pCPD2-Luc, and pCPD3-Luc plasmids were generous gifts from Dr. Chung-ming Chang (National Health Research Institutes, Taiwan). The pPGC-1α plasmid was a generous gift from Dr. Chin-Wen Chi (National Yang-Ming University, Taiwan). To construct the pG6Pase-Luc plasmid, an 800-bp (from-826 to +7 bp) DNA fragment containing the hG6Pase gene promoter was cloned into the pGL3-Basic vector (Promega).

### Nucleocytoplasmic protein extraction and Western blotting

Cells were lysed using the NE-PER Nuclear and Cytoplasmic Extraction Reagents kit (Thermo Scientific, USA). Protein concentrations were measured using the Bradford method. Cell lysates were separated by SDS–PAGE followed by Western blot analysis. The signals of the secondary antibodies were visualized by adding HRP substrate peroxide solution/luminol reagents (Immobilon™ Western Chemiluminescent Substrate, Millipore; mixed at a 1:1 ratio) and detected by the Fujifilm LAS4000 luminescent image analysis system. The following primary antibodies were used: anti-PDH, anti-p-PDH, anti-PDHK, anti-PKM2, anti-HK2, anti-p-AMPK anti-AMPK, anti-FOXO1, anti-PGC-1α (Cell Signaling); anti-ACC, anti-FASN, anti-PPARγ (GeneTex); anti-B23, anti-HNF4α (Santa Cruz Biotechnology). All of the antibodies were used at a 1:1000 dilution.

### Quantification of HBsAg

Cells were seeded in 24-well plates at a density of 8 × 10^4^ cells/well in DMEM medium containing 10% fetal calf serum. After 48 h of incubation, Hep3B/T2 cells were washed twice with phosphate-buffered saline (PBS), pH 7.4, and treated with various concentrations of drugs in serum-free DMEM for 48 h. The amount of HBsAg production in the culture medium was determined by enzyme immunoassay (EIA) kits (GBC, Taiwan). The viability of cells was determined by the MTT cell proliferation assay.

### Quantitative RT-PCR

Total cytoplasmic RNA was isolated using the Maestrozol RNA extraction kit (Maestrogen, Taiwan). A total of 5 μg of RNA and 0.5 μg of oligo-dT primer was heated for 10 min at 70°C. After cooling on ice, the first strand of cDNA was synthesized by Improm II reverse transcriptase (Promega). Quantitative RT-PCR was performed using Light Cycler Fast Start DNA Master SYRB Green I and the LightCycler real-time PCR instrument (Roche Diagnostics). The PCR cycling program consisted of an initial denaturing step at 95°C for 10 min, followed by 45 amplification cycles at 95°C for 12 s, and 54°C for 20 s. RT-PCR analysis used the following primers: HBV FW: CAGGTCTGTGCCAAGT; HBV Rev: TGCGGGATAGGACAAC; (GenBank accession number: V01460). G6Pase FW: GGGTGTAGACCTCCTGTGGA; G6Pase Rev: GAGCCACTTGCTGATTTCC. PEPCK FW: GGGTGCTAGACTGGATCTGC; PEPCK Rev: GAGGGAGAACAGCTGAGTGG; PGC-1α FW: GTCAC CACCCAAATCCTTAT. PGC-1α Rev: ATCTACTGCCT GGAGACCTT. β-actin FW: CCTCTATGCCAACA CAGTGC; β-actin Rev: CATCGTACTCCTGCTTGCTG.

### Quantitative detection of HBV DNA by real-time light cycler PCR

The standard curve showed a good linear range when 10^1^~10^7^ copies of HBV DNA were used. Cells were seeded in 60-mm culture dishes at a density of 1 × 10^6^ cells/well before being treated with various concentrations of drug in serum-free DMEM for 48 h. For quantification of HBV DNA, viral DNA was extracted from culture media using the High Pure Viral Nucleic Acid Kit (Roche, Mannheim, Germany) according to the manufacturer's instructions.

### Sulforhodamine B (SRB) assay

Cells were seeded into plates and cultured over night at 37°C in a 5% CO_2_ incubator. The cells were then treated with GP and HH-F3 for 24 h. The medium was removed immediately after the drug treatment and replaced with 10% precooled trichloroacetic acid (TCA). The cells were fixed for 1 h at 4°C, and the plate was then washed with distilled water and dried. One hundred microliters of a 4-mg/mL solution of SRB (Sigma, St. Louis, MO, USA) in 1% acetic acid was added to each well, and the plate was incubated for 1 h. The plate was then washed five times with 1% acetic acid solution and dried. The SRB in the cells was subsequently dissolved in 150 μL of 10 mM Tris–HCl and measured at 540 nm using an ELISA reader.

### Oil red O assay

For Oil red O staining experiments, Huh7 cells were inoculated at 1 × 10^4^ cells/well in 24-well culture slides and incubated at 37°C overnight. After cells were treated with oleic acid (OA)/palmitic acid (PA) or HH-F3 for 1 day, cells were washed twice with phosphate-buffered saline and fixed with 4% paraformaldehyde. Further processes of Oil Red O staining were performed according to the manufacturer's instructions. After the Oil Red O retained in the cells was extracted with isopropanol, the observance was spectrophotometrically determined at 520 nm.

### Collection of HCC up- and down-regulated gene lists for pathway analysis

Up- and down-regulated gene lists were extracted from the Encyclopedia of Hepatocellular Carcinoma genes Online 3 (EHCO3) database (http://ehco.iis.sinica.edu.tw/) based on majority vote, meaning that the up or down annotation is supported by at least 1 dataset and has at least 33.33% more annotations than its opposite as described previously [29]. The gene lists were used for over-representation analysis for the KEGG pathway database on ConsensusPathDB (http://cpdb.molegen.mpg.de/). Based on the pathway enrichment results, the network diagrams for up- and down-regulated genes were also generated using ConsensusPathDB.

### Statistical analysis

All results were expressed as the mean ± standard error of the mean. Levels of significance were evaluated by two-tailed paired Student's *t*-test or one-way analysis of variance (ANOVA). *P* < 0.05 was considered statistically significant.

## RESULTS

### Pathway analysis of up- and down-regulated genes in hepatocellular carcinoma

**Figure 1:**
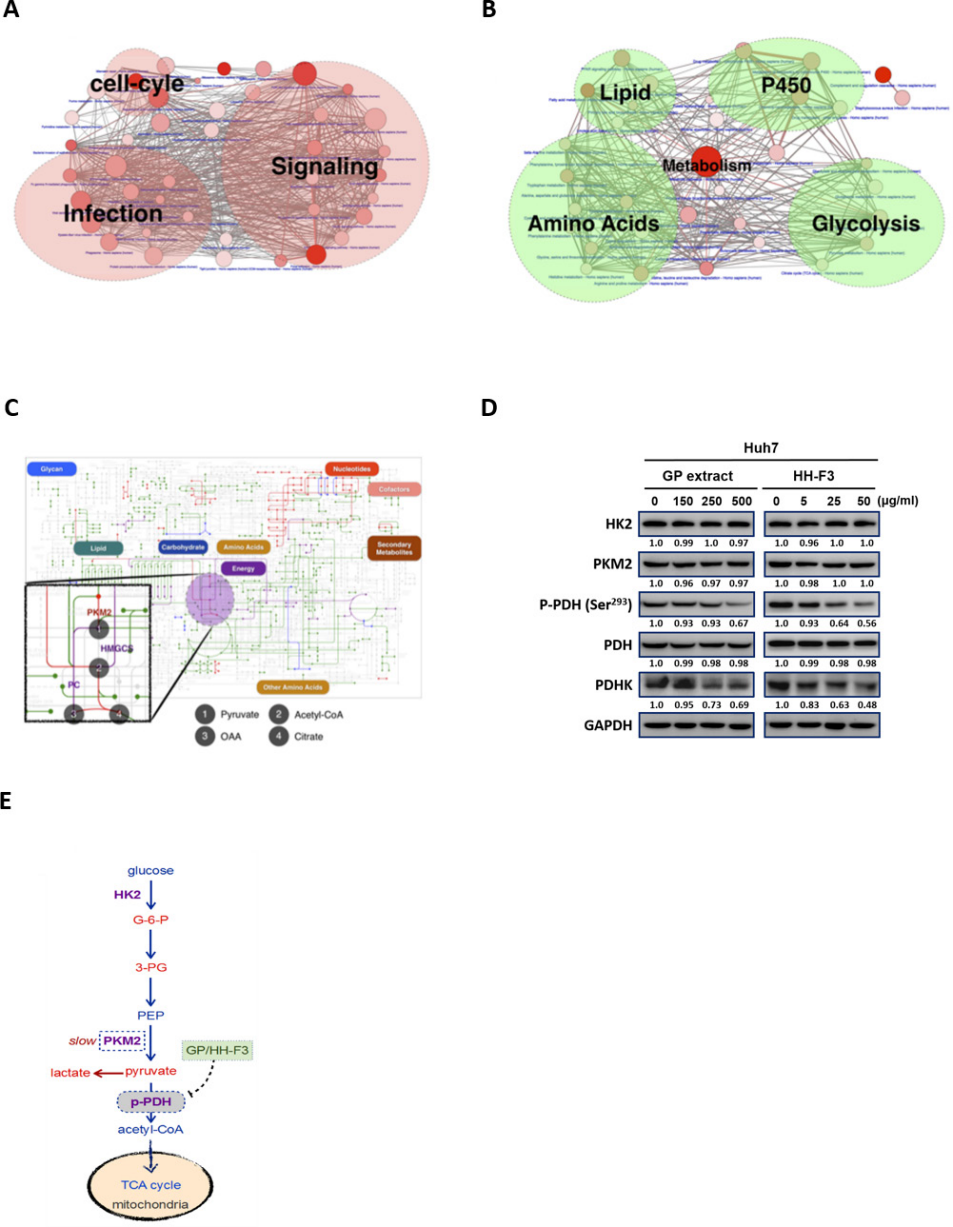
Pathway analysis of up- and down-regulated genes in hepatocellular carcinoma Two network diagrams are presented here to illustrate the pathways enriched by up- and down-regulated genes, respectively. **(A)** Up-regulated genes were mainly enriched in signaling, infection, and cell cycle-related pathways, whereas **(B)** down-regulated genes were enriched only in metabolism pathways related to lipid synthesis, glycolysis, amino acid metabolism, and P450 metabolism. Each node represents a pathway, and the number of genes in the pathway determines the size of the node. The larger the pathway, the bigger the size of the node is. The P-value from pathway enrichment analysis determines the redness of the balls. The lower the *p*-value, the redder the node is. The thickness of the lines between nodes in the network represents the number of genes that the two pathways have in common. **(C)** The blueprint of metabolism pathways adapted from KEGG to illustrate dysregulated reactions based on EHCO3 (green lines: reactions whose enzymes are down-regulated; red lines: reactions whose enzymes are up-regulated). Purple lines are reactions whose enzymes in HCC could be reversed from down-regulation to up-regulation by GP/HH-F3, whereas blue lines are reactions whose enzyme expression levels could be suppressed by GP/HH-F3. **(D)** Huh7 cells were treated with 30% DMSO GP extracts and HH-F3 for 24 h, respectively. Western blot shows the effects after GP/HH-F3 treatment for HK2, PKM2, pyruvate dehydrogenase complex (PDH), phosphorylated PDH (p-PDH) and PDHK with GAPDH used as a control. **(E)** GP/HH-F3 could influence the TCA cycle via down-regulation of phosphorylated PDH, but GP/HH-F3 could not affect other molecules in the TCA cycle. A possible mechanism describing how GP/HH-F3 restores dysregulated glycolysis. Glucose-6-phosphate (G-6-P), 3-phosphoglycerate (3-PG), and phosphoenolpyruvate (PEP) are key intermediates in glycolysis.

Pathway analysis of our collected HCC gene signatures from EHCO3 indicates that up-regulated genes are mainly enriched in signaling, infection and cell cycle-related pathways, whereas down-regulated genes are enriched in metabolism pathways related to lipid synthesis, glycolysis, and amino acid metabolism (Figure 1A–1B). The up-regulated genes are mostly factors modulating signaling pathways in tumor cells to maintain the production of necessary materials for proliferation, and many of them are drug targets such as mitogen-activated protein kinase (MAPK). On the other hand, the down-regulated genes are mainly involved in lipid, amino acids, cytochrome P450, and glycolysis-related pathways. Down-regulation of enzymes might implicate that some metabolic reactions have been slowed down and resulted in the accumulation of upstream intermediates, such as glucose-6-phosphate (G-6-P) and 3-phosphoglycerate (3-PG), which could be used by pathways needed for HCC to proliferate [30, 31].

Recent studies also support that metabolic reprogramming is the core hallmark of cancer, and one of the functions of activated oncogenes and inactivated tumor suppressors is to reprogram cellular metabolism [30]. Here, we employed the blueprint of metabolism pathways adapted from KEGG to illustrate dysregulated reactions based on EHCO3. As described, treatment with GP could reverse most metabolism-regulated gene signatures in a DMN-induced rat liver fibrosis model [22]. Using these GP gene signatures, bioinformatics analysis predicts that GP might modulate some of the dysregulated metabolic enzymes (Figure 1C). We next tested whether GP and its purified active fraction HH-F3 might affect glycolysis. Huh7 cells were treated with various concentrations of GP and HH-F3 for 24 h. Western blot analysis indicated that GP and HH-F3 did not change the total protein expression of hexokinase 2(HK2) and pyruvate kinase muscle isozyme M2 (PKM2), but suppressed pyruvate dehydrogenase kinase (PDHK) and phosphorylated pyruvate dehydrogenase (PDH) (Ser^293^) (Figures 1D–1E).

### HH-F3 suppresses gluconeogenic enzymes, G6Pase and PEPCK gene expression

**Figure 2:**
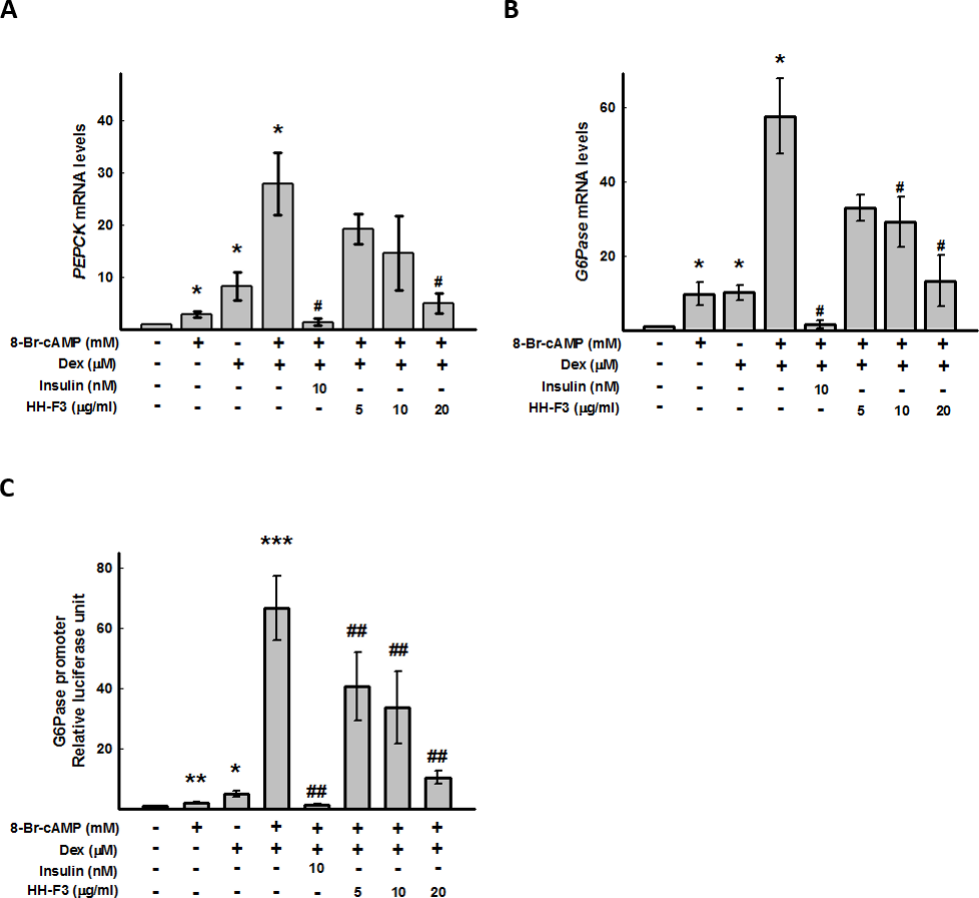
HH-F3 suppresses 8-bromo-cAMP/dexamethasone-induced gluconeogenic enzymes*G6Pase* and *PEPCK* gene expression, and G6Pase promoter activity Human hepatoma Hep3B/T2 cells were pre-treated with 0.5 mM 8-bromo-cAMP (8-Br-cAMP) alone or 0.5 μM dexamethasone (Dex) alone or both for 30 minutes. Different concentrations of HH-F3 were then added in serum-free DMEM for 24 h. The mRNAs of the gluconeogenic genes **(A)** *PEPCK* and **(B)** *G6Pase* were isolated and measured by Q-RT PCR and normalized to β-actin. Insulin was used as a positive control. **(C)** For the promoter activity assay, Hep3B/T2 cells were transfected with luciferase reporter plasmids driven by the G6Pase promoter. The luciferase activities were normalized to the β-galactosidase activity from co-transfected pCMV-β-galactosidase plasmids. *P < 0.05 compared with the vehicle group. #*P* < 0.05, ##*P* < 0.01 compared with the 8-Br-cAMP/Dex-induced group (*n* = 3).

To examine the effects of GP and HH-F3 on gluconeogenesis in HCC, the Hep3B/T2 hepatoma cell line expressing HBV was pretreated with 8-Bromo-cAMP and dexamethasone (8-Br-cAMP/Dex) for 30 min and then was treated with HH-F3 for 24 h. All of the reagents used, including 8-Br-cAMP, dexamethasone, and HH-F3 (20 μg/ml), did not affect cell viability within 24 h (Supplementary Figure 1). Consistent with previous studies, 8-Br-cAMP/Dex could synergistically activate gene expression of key gluconeogenic genes, including *phosphoenol pyruvate carboxykinase (PEPCK)* and *glucose-6-phosphatase (G6Pase),* in Hep3B/T2 cells. Treatment with HH-F3 suppressed 8-Br-cAMP/Dex-induced *PEPCK* and *G6Pase* gene expression dose-dependently (Figure 2A and 2B). Similarly, the incubation of Hep3B/T2 cells with 8-Br-cAMP/Dex increased G6Pase promoter activity. Exposure to HH-F3 significantly reduced 8-Br-cAMP/Dex-induced G6Pase promoter activity (Figure 2C). These results indicate that HH-F3 can inhibit key gluconeogenic enzyme gene expression.

### HH-F3 suppresses gluconeogenic coactivator PGC-1α expression

**Figure 3:**
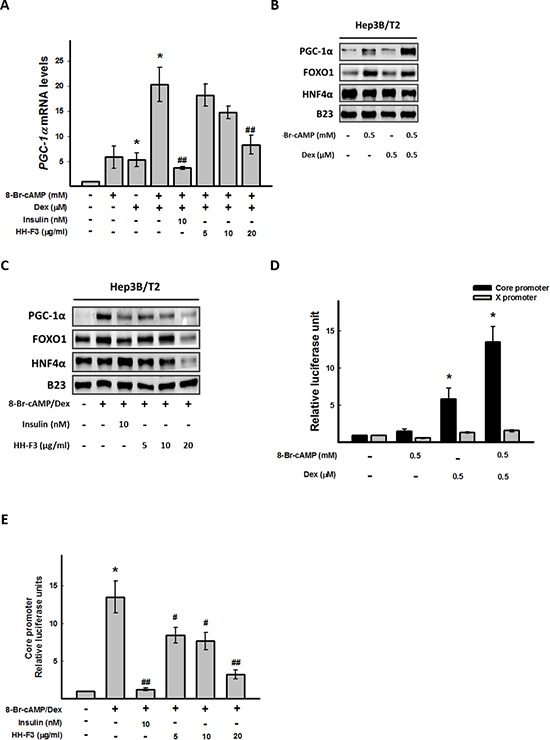
HH-F3 suppresses 8-Br-cAMP/dexamethasone-induced coactivator transcription factor gene expression and HBV core promoter activity Hep3B/T2 cells were pretreated with 8-Br-cAMP/Dex for 30 min. Different concentrations of HH-F3 were then added in serum-free DMEM for 24 h. **(A)** *PGC-1α* mRNA was measured by Q-RT PCR and normalized to β-actin. Insulin was used as a positive control. **(B and C)** Nuclear extracts were isolated, and the levels of PGC-1α and HNF-4α, FOXO1 proteins were determined by Western blotting. B23 was used as a nuclear protein loading control. **(D and E)** For the promoter activity assay, Hep3B/T2 cells were transfected with luciferase reporter plasmids driven by the HBV core promoter (CP) and HBV X promoter (XP). One day after 8-Br-cAMP/Dex treatment in the absence or presence of different concentrations of HH-F3 in serum-free DMEM, cell lysates were prepared for luciferase activity analysis as described previously. *P < 0.05 compared with the vehicle group. #*P* < 0.05, ##*P* < 0.01 compared with the 8-Br-cAMP/Dex-induced group (*n* = 3).

The metabolic regulator PGC-1α robustly coactivates the transcription of gluconeogenic enzyme genes through HNF4α and FOXO1 [16, 32]. To determine whether HH-F3 might affect the coactivation of PGC-1α, HNF4α and FOXO1 to regulate gluconeogenic enzyme transcription, Hep3B/T2 cells were pretreated with 8-Br-cAMP/Dex for 30 min followed by treatment with HH-F3 for 24 h, and then the level of PGC-1α, HNF4α and FOXO1 were examined by Q-RT PCR or Western blot analysis. As shown in Figure 3A and 3B, 8-Br-cAMP/Dex synergistically activated the gene and protein expression of PGC-1α. Treatment with HH-F3 suppressed 8-Br-cAMP/Dex-induced *PGC-1α* gene expression (Figure 3A and 3C). HH-F3 also decreased the protein levels of FOXO1 and HNF4α, which were associated with gluconeogenic transcription factor expression in Hep3B/T2 cells (Figure 3C). These results suggest that HH-F3-suppressed gluconeogenic enzyme expression may occur via inhibition of *PGC-1α* gene expression.

### HH-F3 inhibits 8-Br-cAMP/Dex-induced core promoter expression

PGC-1α has been shown to coactivate FOXO1 and HNF4α to regulate HBV gene transcription [15, 33]; therefore, the activities of HBV core and X promoters were estimated upon 8-Br-cAMP/Dex treatment in the absence or presence of HH-F3. Interestingly, 8-Br-cAMP/Dex synergistically activated hepatitis B viral core promoter activity, but had no effect on the HBV X promoter (Figure 3D). Treatment with HH-F3 could dose dependently suppress 8-Br-cAMP/Dex-induced HBV core promoter activity in Hep3B/T2 cells (Figure 3E).

### Overexpression of PGC-1α reverses the HH-F3-mediated decrease of HBV core promoter activity

**Figure 4:**
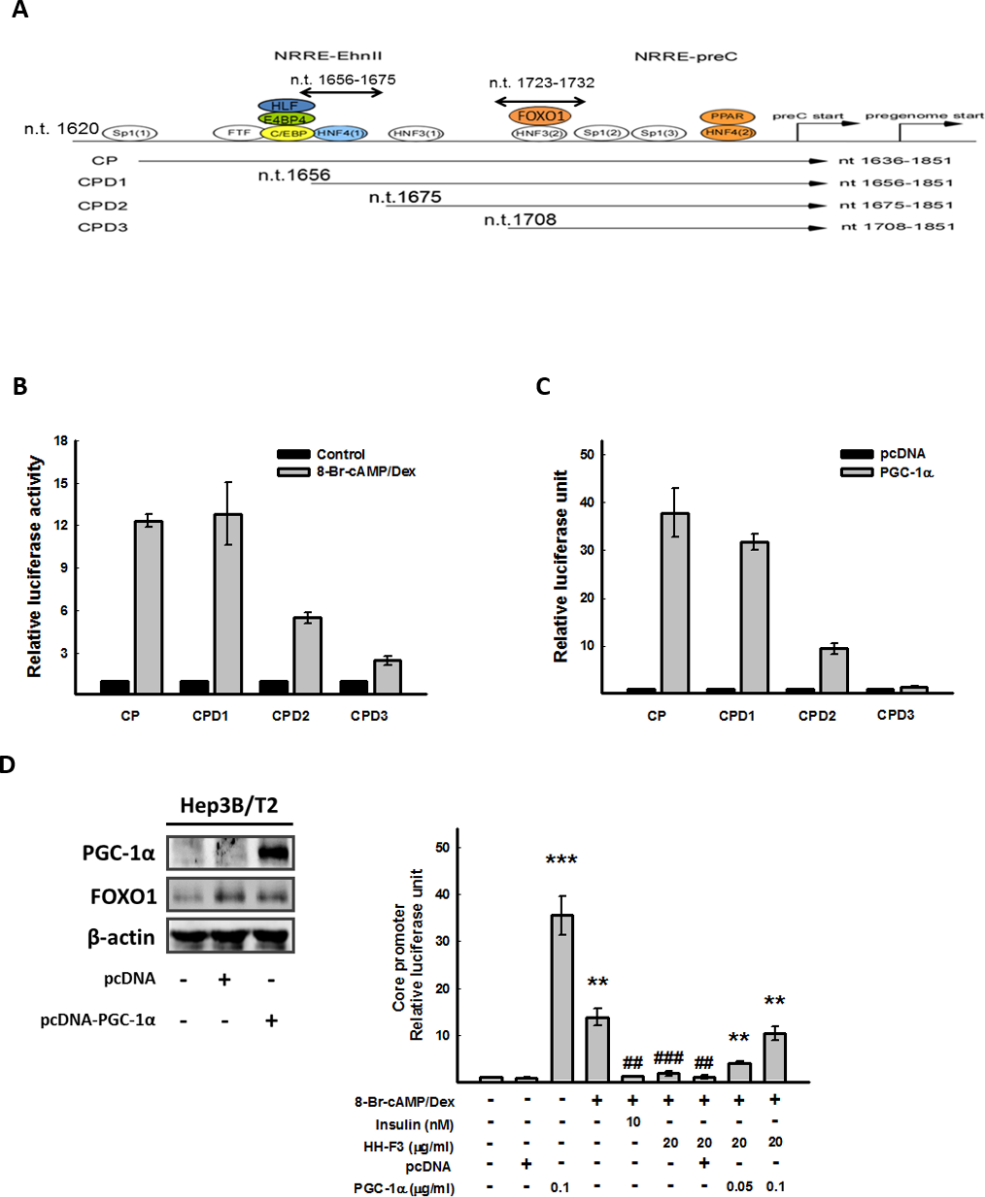
CP (nt 1656–1675) is the cis-element responsible for 8-Br-cAMP/Dex to HBV core promoter activity, and overexpression of PGC-1α can reverse the inhibition of HH-F3 to the core promoter **(A and B)** Hep3B/T2 cells were transfected with luciferase reporter plasmids driven by the HBV core promoter (CP) and three truncated HBV core promoters (CPD1, CPD2 and CPD3), respectively. One day after 8-Br-cAMP/Dex treatment, cell lysates were prepared for luciferase activity analysis. **(C)** Various HBV core promoter constructs were cotransfected with pcDNA-PGC-1α, respectively, and then were subjected to the luciferase assay. **(D)** HBV core promoter (CP) was cotransfected with pcDNA-PGC-1α. One day after 8-Br-cAMP/Dex treatment in the absence or presence of different concentrations of HH-F3 in serum-free DMEM, cell lysates were prepared for the luciferase assay. Hep3B/T2 cells were transfected with pcDNA-PGC-1α (0.1 μg/ml), and PGC-1α protein levels were determined by Western blot analysis. β-actin was used as a loading control. **P < 0.01, ***P < 0.005 compared with the vehicle group. ##*P* < 0.01, ###*P* < 0.005 compared with the 8-Br-cAMP/Dex-induced group (*n* = 3).

The combination of 8-Br-cAMP and Dex could synergistic stimulate HBV core promoter activity (Figure 3D); thus, to identify the 8-Br-cAMP/Dex response region located within the HBV core promoter, Hep3B/T2 cells were transfected with full-length and truncated HBV core promoter constructs (CP and CPD1–3) (Figure 4A), and then exposed to 8-Br-cAMP/Dex. The luciferase assay indicated that the CP (nt 1636–1851) and CPD1 (nt 1656–1851) exhibited the maximum luciferase activity, which was much higher than that of CPD2 (nt 1675–1851) and CPD3 (nt 1708–1851). When nt 1656–1675 of CPD1 was deleted, the 8-Br-cAMP/Dex-induced luciferase activity was significantly reduced (Figure 4B). It has been reported that PGC-1α can regulate HBV gene expression in HBV transgenic mice [33]. To address whether PGC-1α was involved in HBV core promoter activation, full-length and truncated HBV core promoter constructs (CP and CPD1–3) were cotransfected with the plasmid encoding human PGC-1α into Hep3B/T2 cells. As expected, when nt 1656–1675 of CPD1 were deleted (Figure 4B), luciferase activity was significantly reduced (Figure 4C). These results suggest that the sequence spanning nt 1656 to nt 1675 within the HBV core promoter might be important for PGC-1α to induce HBV core promoter activity. Our above results showed that HH-F3 inhibited PGC-1α mRNA and protein expression, as well as HBV core promoter activity (Figure 3). Next, the HBV core promoter was cotransfected with PGC-1α in the presence or absence of 8-Br-cAMP/Dex in Hep3B/T2 cells, and the levels of PGC-1α and FOXO1 were determined (Figure 4D, left panel). The luciferase assay indicated that overexpression of PGC-1α effectively reversed the HH-F3-triggered HBV core promoter inhibition (Figure 4D, right panel). These results suggest that HH-F3-suppressed HBV core promoter activation occurred through the inhibition of gluconeogenic coactivator PGC-1α expression.

### HH-F3 suppresses gluconeogenic enzyme expression, HBV surface antigen (HBsAg) gene expression, and the HBV DNA level in Hep3B/T2 and 1.3ES2 cells

**Figure 5:**
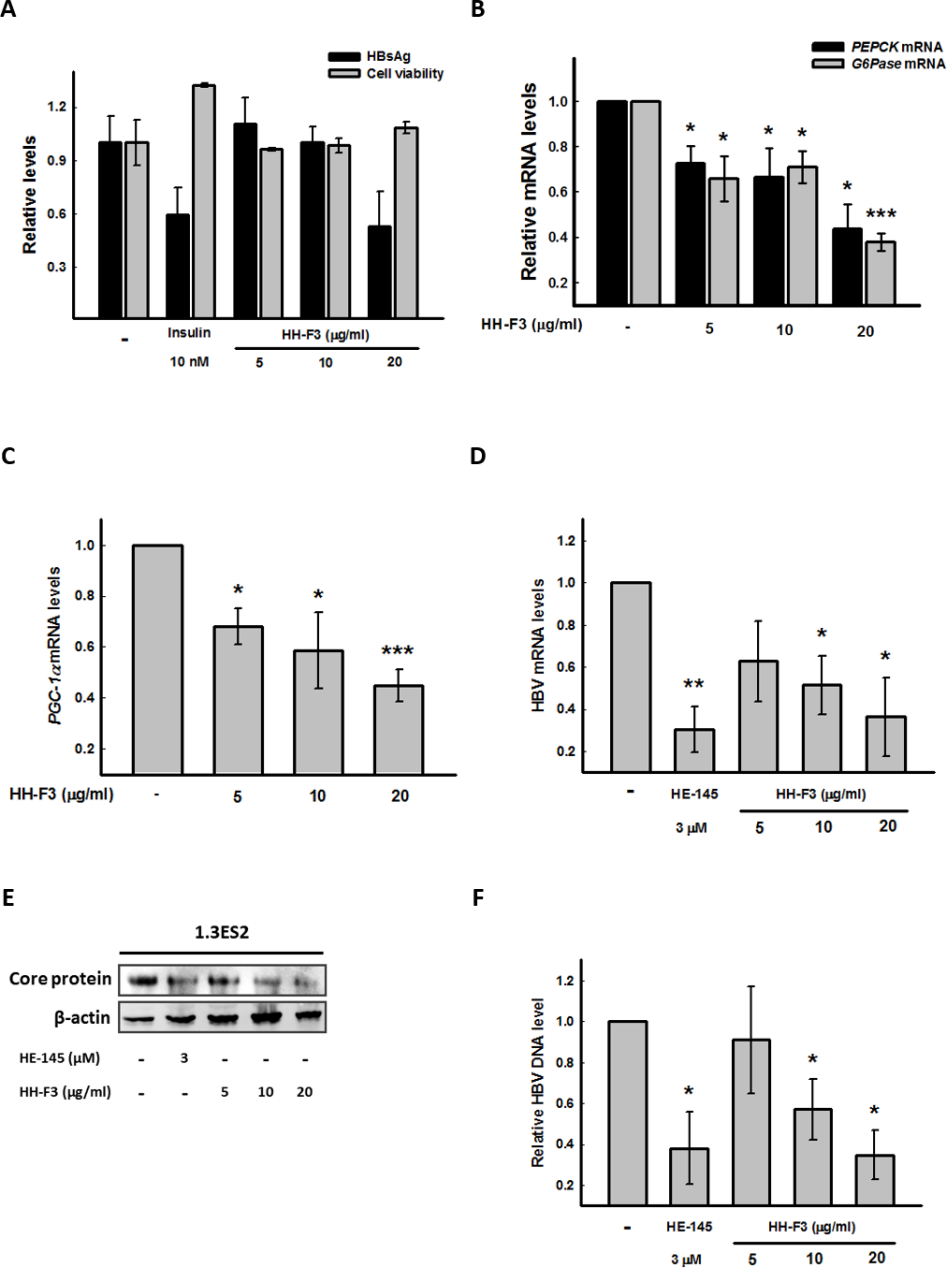
HH-F3 suppresses HBsAg, gluconeogenic enzyme gene expression, and the HBV DNA level in Hep3B/T2 and 1.3ES2 cells **(A)** Hep3B/T2 cells were treated with different concentrations of HH-F3 in serum-free DMEM medium for 48 h. HBsAg was determined by ELISA. Insulin was used as a positive control. **(B–D)** Cultured 1.3ES2 cells were treated with different concentrations of HH-F3 in serum-free DMEM medium for 48 h. The gluconeogenic enzyme genes*G6Pase, PEPCK,* and *PGC-1α*, as well as HBV mRNA expression, were measured by Q-RT PCR and normalized to β-actin. **(E)** HBV core protein levels were determined by Western blot analysis. **(F)** Q-RT PCR was used to detect wild-type HBV DNA in the medium of 1.3ES2 cells. *P < 0.05, **P < 0.01, ***P < 0.005 compared with the vehicle group (*n* = 3).

To evaluate whether HH-F3 might affect HBV gene expression, we used the Hep3B/T2 cell line, which can continuously secrete HBsAg into the culture medium, as our cell model. Treatment of HH-F3 for 48 h resulted in a dose-dependent suppression of HBsAg expression in Hep3B/T2 cells (Figure 5A). Next, we used the 1.3ES2 cell line, which is a subline of HepG2 cells, containing 1.3 copies of the entire HBV genome stably integrated into the HepG2 cell genome [34]. To explore whether HH-F3 could inhibit HBV gene expression via the regulation of PGC-1α, 1.3ES2 cells were treated with HH-F3 for 48 h. As a result, HH-F3 not only suppressed gluconeogenic enzyme *G6Pase, PEPCK* (Figure 5B) and coactivator *PGC-1α* gene expression (Figure 5C) but also suppressed HBV mRNA (Figure 5D) and core protein levels (Figure 5E) in 1.3ES2 cells. Additionally, treatment with HH-F3 significantly reduced HBV DNA replication in a dose-dependent manner in 1.3ES2 cells (Figure 5F). The synthetic compound HE-145 has been reported to inhibit HBV gene expression [35]; therefore, it was used as a positive control. These results indicate that HH-F3 inhibition of viral expression may be associated with gluconeogenesis machinery.

### HH-F3 treatment decreases fatty acid synthesis and lipid accumulation in HCC

**Figure 6:**
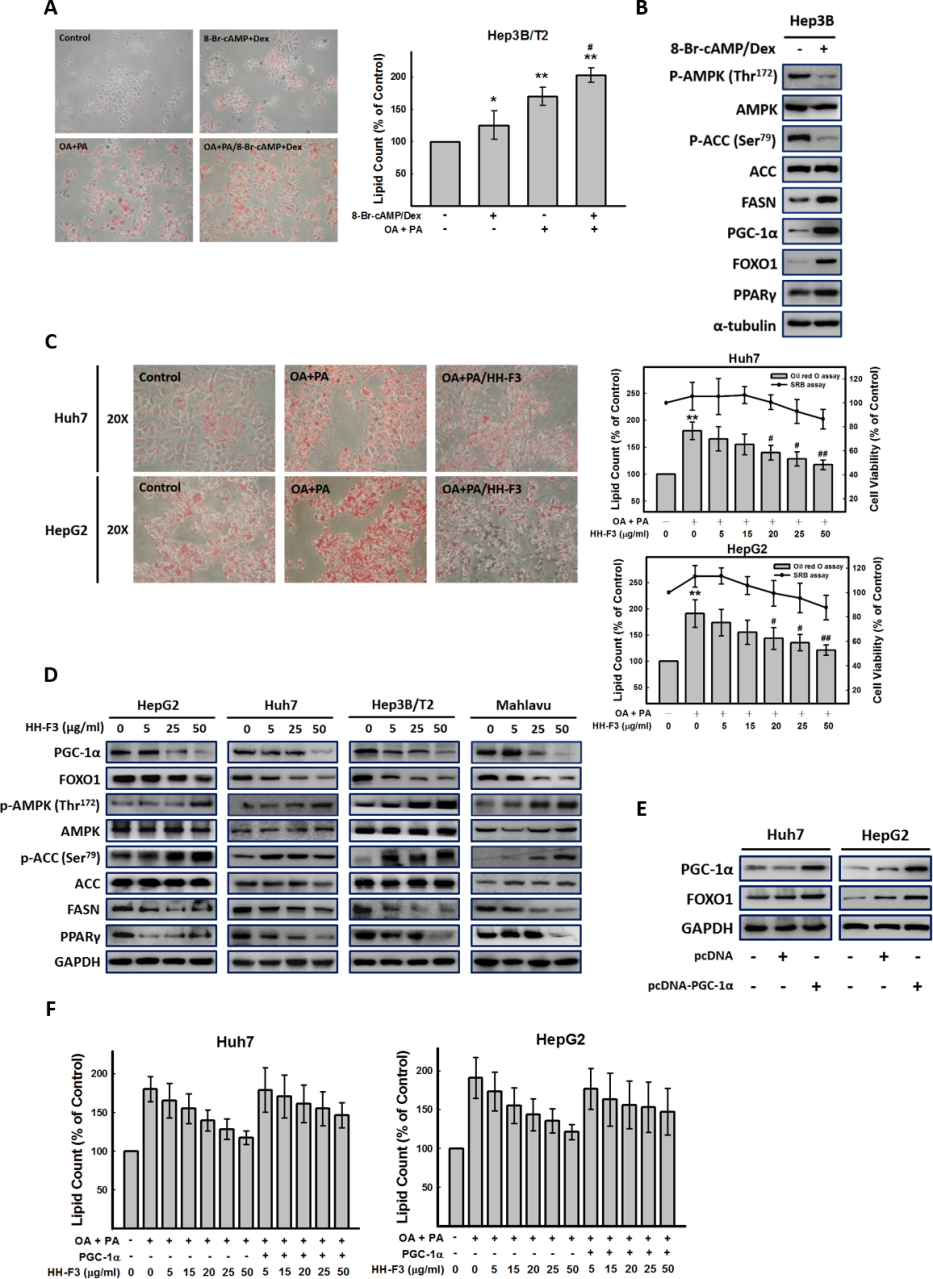
HH-F3 treatment decreases fatty acid synthesis in HCC **(A)** Hep3B/T2, Huh7 and HepG2 cells were incubated with 8-Br-cAMP/Dex and oleic acid (OA) (1 mM) and palmitic acid (PA) (2 mM) for 24 h, respectively. Intracellular lipid accumulation could be visualized by Oil red O staining, and the amount of lipid in each treatment was calculated. **(B)** Cultured human hepatoma Hep3B/T2 cells were treated with 8-Br-cAMP/Dex for 24 h, and then were subjected to Western blot analysis (*n* = 3). **(C)** Incubation of Huh7 and HepG2 cells with OA and PA for 24 h resulted in significant intracellular lipid accumulation as visualized by Oil red O staining. Cells were treated with HH-F3 for 24 h, and the lipid content and cell viability were determined (OA/PA/HH-F3: OA/PA and HH-F3 co-treatment). **(D)** HepG2, Huh7, Hep3B/T2, and Mahlavu cells were treated with HH-F3, and then were subjected to Western blot analysis (*n* = 3). **(E)** pcDNA-PGC-1α was transfected into Huh7 and HepG2 cells, and the protein levels of PGC-1α and FOXO1 were measured. **(F)** pcDNA-PGC-1α-transfected Huh7 and HepG2 cells were incubated with OA/PA in the absence or presence HH-F3 for 24 h, and intracellular lipid accumulation was counted. *P < 0.05, **P < 0.01 compared with the control group. #*P* < 0.05, ##*P* < 0.01 compared with the OA/PA-induced group.

AMPK is a cellular energy sensor that inhibits ATP consumption and stimulates ATP production under energy-depleted conditions. Activated AMPK can stimulate ATP generation by increasing fatty acid oxidation and reducing ATP hydrolysis through decreased lipogenesis and gluconeogenesis. It has been reported that activated AMPK can phosphorylate TORC2, which mediates CREB-dependent transcription of *PGC-1α* and *PGC-1α* downstream targets *PEPCK* and *G6Pase*, thus inhibiting hepatic gluconeogenesis [36, 37]. We next examined whether HH-F3 could induce the phosphorylation of AMPK in Hep3B/T2 cells. In Figure 6D and Supplementary Figure 2A, treatment of Hep3B/T2 cells with HH-F3 resulted in the phosphorylation of AMPK and ACC in a dose-dependent manner. Moreover, the AMPK inhibitor compound C could partially reverse the HH-F3-suppressed G6Pase promoter activity (Supplementary Figure 2B), suggesting that HH-F3 may act through the activation of AMPK to suppress G6Pase expression. Furthermore, incubation of Hep3B/T2 cells with 8-Br-cAMP/Dex for 24 h significantly increased lipid accumulation (Figure 6A) and lipogenesis-related protein expression such as ACC and FASN expression (Figure 6B). Previous reports have shown that lipogenesis is regulated by the AMPK/PGC-1α signaling axis [17, 38]. These findings prompted us to examine whether GP extracts and HH-F3 might modulate lipogenesis. Our results show that treatment with GP extracts and HH-F3 decreased lipid accumulation (Supplementary 3B and Figure 6C). GP extracts and HH-F3 suppressed the activity of fatty acid (FA) synthesis by activating AMPK (increased AMPK phosphorylation) and inhibited PGC-1α expression in HepG2, Huh7 and Mahlavu cells (Figure 6D and Supplementary Figure 3A). Moreover, GP extracts and HH-F3 treatment leads to increased phosphorylation and consequent inactivation of the AMPK downstream target ACC, as well as reduced the protein level of FASN in a dose-dependent manner. These results indicate that GP extract and HH-F3 treatment may decrease fatty acid synthesis in HCC. Next, we explored whether PGC-1α was involved in HH-F3-mediated lipogenesis in Huh7 and HepG2 cells. As shown in Figures 6E and 6F, overexpression of PGC-1α could effectively reverse the HH-F3-mediated inhibition of lipid deposition.

## DISCUSSION

The present study showed the following findings: (I) GP extracts and its active fraction HH-F3 suppress 8-Br-cAMP/Dex-induced gluconeogenic enzyme gene expression; (II) HH-F3 inhibits the expression of 8-Br-cAMP/Dex-induced gluconeogenic coactivator PGC-1α and transcription factors FOXO1 and HNF4α; (III) HH-F3 inhibits HBV core promoter activation, HBV gene expression, and HBV DNA replication in Hep3B/T2 and 1.3ES2, which are HBV-containing cell lines; and (IV) HH-F3 treatment decreases fatty acid synthesis and lipid accumulation in HCC cells.

The bulk of ATP used by various types of cells to maintain homeostasis is produced by the oxidation of pyruvate in the tricarboxylic acid cycle (TCA cycle). The fate of pyruvate depends on the cell energy charge. In the liver, intestine, and kidney, if the energy charge is high, pyruvate is directed toward gluconeogenesis. However, when the energy charge is low, pyruvate is preferentially oxidized to CO_2_ and H_2_O in the TCA cycle. Proliferating cells, such as tumor cells, facilitate their macromolecular synthesis pathways through expressing an alternative PKM2, so that the intermediates could branch off glycolysis. The TCA cycle consumes acetyl-CoA and water, reduces NAD^+^ to NADH, and produces carbon dioxide as a waste byproduct. The NADH generated by the TCA cycle is fed into the oxidative phosphorylation (electron transport) pathway. The net result of these two closely linked pathways is the oxidation of nutrients to produce usable chemical energy in the form of ATP. Meanwhile, by phosphorylating the pyruvate dehydrogenase complex (PDHc), tumor cells tend to further suppress the downstream TCA cycle, electron transport chain (ETC), and pro-apoptotic mediators such as cytochrome c and apoptosis-inducing factors in the mitochondria [39]. In humans, PDHc activity is inhibited in response to site-specific phosphorylation at three sites on PDH (Ser^232^, Ser^293^, and Ser^300^) [40]. The activity of PDH regulation via phosphorylation is currently implicated in the altered patterns of metabolism in cancer, obesity and insulin resistance [41]. Here, we found that HH-F3 could inhibit PDHK and phosphorylation of PDH (Figure 1D), suggesting that HH-F3 might modulate glucose metabolism in cells.

Excessive hepatic glucose production, a characteristic of diabetes mellitus due to insulin deficiency/resistance and elevated glucagon levels, is responsible for the fasting hyperglycemia in diabetes. Insulin counteracts the action of glucagon on hepatic gluconeogenesis mainly at the transcriptional level through the transcriptional coactivator PGC-1α [32]. Glucose is converted into fatty acids in the liver through a process called lipogenesis, which is also regulated by PGC-1α. This process is increased in people who have diabetes, hepatitis, fatty liver disease and cancer. PGC-1α coordinately regulates mitochondrial and fatty acid metabolism to promote tumor growth [17]. Recent studies have indicated that metabolic drugs, such as metformin [42] or dichloroacetate (DCA) [39], might reduce the risk of HCC. Metformin reduces the level of glucose and inhibits fatty acid synthesis. Treatment with metformin may result in cancer cells with no energy to proliferate and then prevent tumors from developing [43]. Similarly, HH-F3 inhibits key gluconeogenic enzymes and lipogenesis-related proteins via the suppression of PGC-1α (Figure 2 and Figure 6). These results implicate that HH-F3 might regulate the action of glucagon on hepatic gluconeogenesis, fatty acid synthesis and lipid accumulation mainly through the PGC-1α signaling pathway.

The HBV genome is a 3.2-kb partially double-stranded circular DNA with four open reading frames that encode viral core antigen, viral DNA polymerase, viral surface antigen (HBsAg), and X protein. During HBV replication, the 3.5-kb mRNA not only serves as the template for reverse transcription but also encodes the viral core protein and HBV DNA polymerase [44]. HBV-specific protein, RNA, and DNA are all involved in the process of viral replication. It has been reported that mutations in core promoter sequences frequently occurs. A core promoter mutation would increase the ability of viral replication and further increase the risk of liver cancer [7]. In the present study, we found that HH-F3 could suppress viral core protein expression and core promoter activity and could decrease virus replication via the inhibition of the metabolism regulator PGC-1α (Figure 3–5). Consistently, previous reports have shown that PGC-1α is a critical determinant in both gluconeogenesis and HBV biosynthesis, suggesting that the regulation of viral transcription and metabolic gene expression might utilize similar hepatic signal transduction pathways [32, 33, 45].

Lipids have been linked to many pathological processes, including hepatic steatosis (fatty liver), which manifests as an excess accumulation of lipids in hepatocytes. Abnormal lipid accumulation is associated with multiple genetic defects in energy metabolism and liver malignancy. Liver cancer cells require more energy for survival through dysregulated *de novo* lipogenesis, which may contribute to liver oncogenesis and human HCC [46]. In HCC, the extent of aberrant lipogenesis correlates with clinical aggressiveness, activation of the AKT-mTOR signaling pathway, and suppression of AMPK [47]. In addition, the protein expression of FASN, a key enzyme of lipogenesis that is overexpressed in HCC, is known to be negatively regulated by AMPK. Our previous study demonstrated that HH-F3 can inhibit PTEN/PI3K/AKT pathways in HCC cells. In the current study, we found that HH-F3 inhibited FASN protein expression and activated the AMPK pathway in HCC cells (Figure 6 and Supplementary Figure 2). HH-F3 modulated the expression of lipogenesis-related proteins and gluconeogenic enzyme gene expression via AMPK and in a PGC-1α-dependent manner. The AMPK inhibitor compound C could partially reverse HH-F3-suppressed G6Pase promoter activity and lipid accumulation (Supplementary Figure 2B and 2D). However, compound C could not reverse the inhibition of HH-F3 at the core promoter in Hep3B/T2 cells (Supplementary Figure 2C). These results indicate that HH-F3 regulates HBV gene expression and replication only through PGC-1α signaling.

In previous studies, mTORC1 could increase mitochondrial DNA content and the expression of genes involved in oxidative metabolism through mediating PGC-1α and the transcription factor Ying-Yang 1 (YY1), which regulate mitochondrial biogenesis and oxidative function. mTOR inhibitor rapamycin decreased the gene expression of the mitochondrial transcriptional regulators PGC-1α in skeletal muscle cells [48]. However, some reports showed that the activation of the mTOR-signaling pathway could inhibit HBV RNA transcription and DNA replication [49, 50]. Recent report also indicated that lower concentration (~200 nM) of rapamycin could enhance HBV production, but higher concentration (more than 200 nM) resulted in decrease HBsAg level [51]. To evaluate whether rapamycin might affect HBV gene expression through PGC-1α, we used the Hep3B/T2 cell line, which can continuously secrete HBsAg into the culture medium, as our cell model. Supplementary Figure 4A showed that rapamycin only decreased PGC-1α expression, but not FOXO1 and HNF4α expression in HCC cell lines. In addition, rapamycin only slightly decreased HBsAg expression in Hep3B/T2 cells (Supplementary Figure 4B). In our study, HH-F3 could regulate HBV replication through down-regulation of PGC-1α, FOXO1, and HNF4α. Consequently, HH-F3 is a better HBV inhibitor than rapamycin.

In summary, GP extract and its active fraction HH-F3 not only suppressed gluconeogenesis and lipogenesis but also inhibited HBV gene expression and DNA replication through a PGC-1α-dependent pathway in HCC cells. Targeting PGC-1α may be a novel therapeutic strategy for HBV infection. Together, our results indicate that HH-F3 might be a potent agent for the treatment of patients with chronic hepatitis B associated with metabolic syndrome.

## SUPPLEMENTARY FIGURE AND TABLE


