# Supplementary material for: Gluconeogenesis, lipogenesis, and HBV replication are commonly regulated by PGC-1α-dependent pathway
Source: Oncotarget. 2015 Feb 3;6(10):7788–803. doi: 10.18632/oncotarget.3050 (PMC4480716; doi:10.18632/oncotarget.3050)
Supplement: Supplementary file 1 [file oncotarget-06-7788-s001.pdf]

SUPPLEMENTARY FIGURES

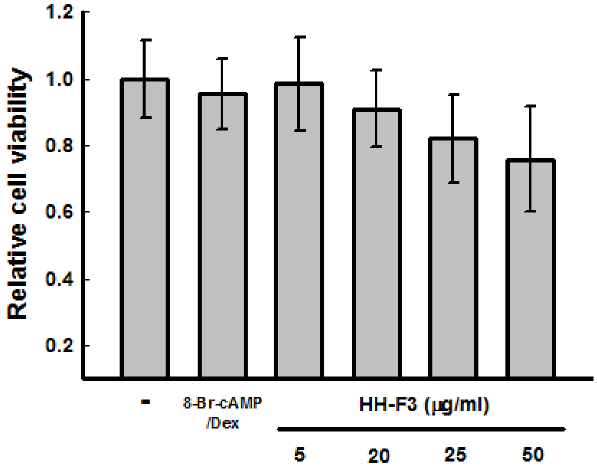

Supplementary Figure S1: The effect of HH-F3 on cell viability in Hep3B/T2 cells. Hep3B/T2 cells were treated with different concentrations of HH-F3 in serum-free DMEM for 24 h. Cell viability was determined by the MTT assay.

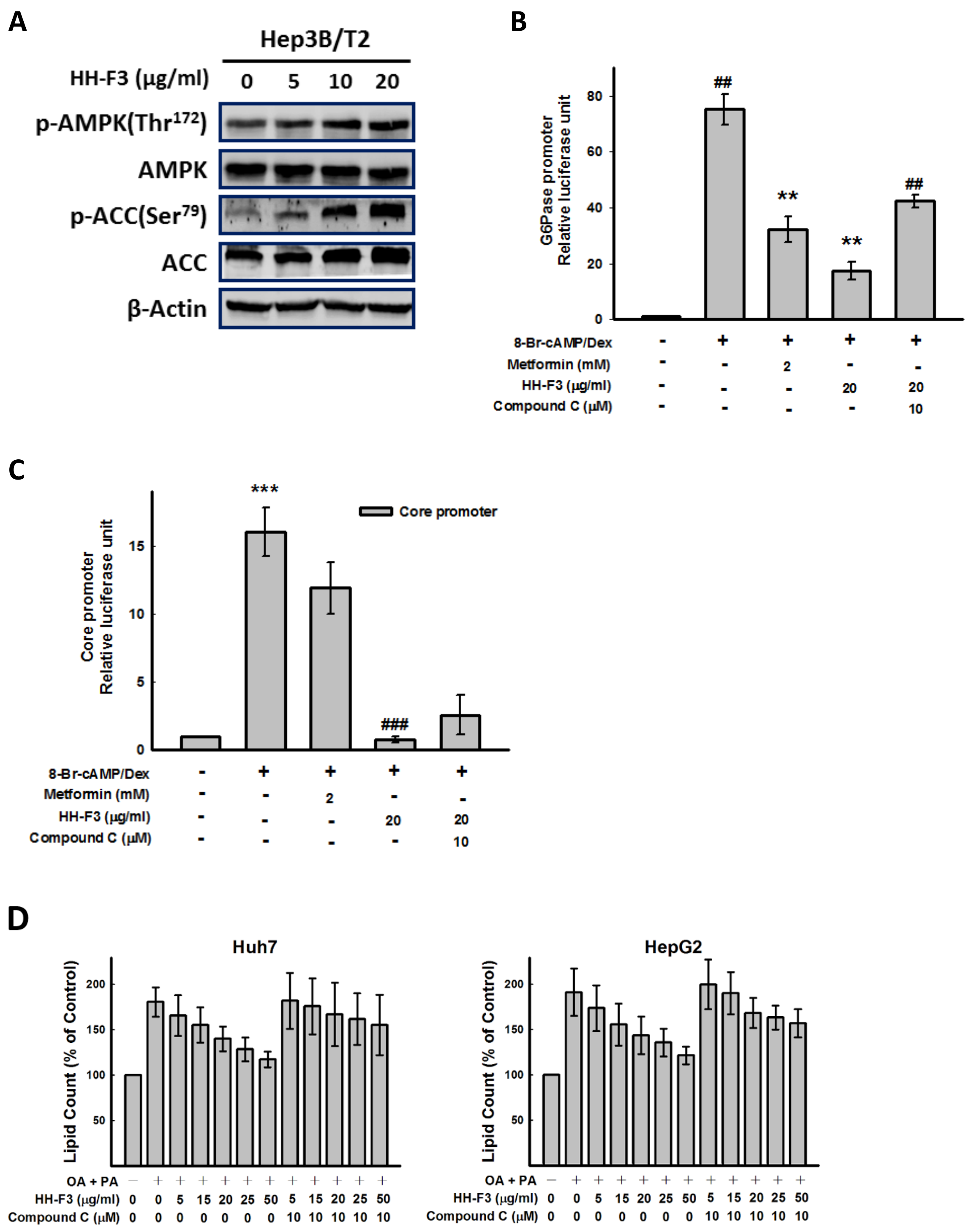

Supplementary Figure S2: HH-F3 may act through the activation of AMPK to suppress gluconeogenic enzyme gene expression. Hep3B/T2 cells were pretreated with 8-Br-cAMP/Dex for 30 min, followed by the addition of different concentrations of HH-F3 in serum-free DMEM for 24 h. **(A)** Cell extracts were subjected to Western blotting. **(B)** For the promoter activity assay, Hep3B/T2 cells were transfected with luciferase reporter plasmids driven by the glucose-6-phosphatase promoter. 8-Br-cAMP/Dex stimulated G6Pase promoter activities were assayed in the absence or presence of either HH-F3 or HH-F3 with the AMPK inhibitor Compound C. One day after treatment, the cell lysates were prepared for luciferase activity analysis as described previously. Metformin was used as an AMPK positive control. **(C)** 8-Br-cAMP/Dex-stimulated Core promoter activities were assayed in the absence or presence of either HH-F3 or HH-F3 with the AMPK inhibitor Compound C. One day after treatment, cell lysates were prepared for luciferase activity analysis. **(D)** OA- and PA-stimulated intracellular lipid accumulation in Huh7 and HepG2 cells was assayed in the absence or presence of either HH-F3 or HH-F3 with the AMPK inhibitor Compound C. $^{**}P < 0.01$, $^{***}P < 0.005$ compared with the vehicle group. $^{\#\#}P < 0.01$, $^{\#\#\#}P < 0.005$, compared with the 8-Br-cAMP/Dex-induced group.

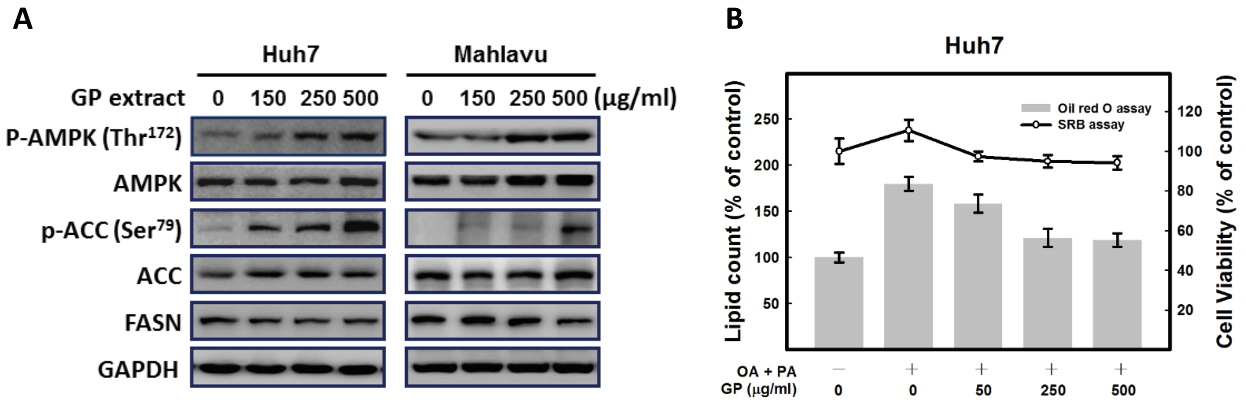

Supplementary Figure S3: GP extract suppresses lipogenesis. **(A)** Huh7 and Mahlavu cells were treated with 30% DMSO GP extract, and then subjected to western blot analysis using antibodies against phospho-AMPK, phospho-ACC (Ser^{79}), AMPK, ACC, and FASN (n = 3). **(B)** Incubation of Huh7 cells with OA and PA for 24 h resulted in a significant intracellular lipid accumulation as visualized by Oil red O staining Cell viability was determined by SRB assay. (OA + PA/HH-F3: oleic acid and palmitic acid with HH-F3 co-treatment). Huh7 cells treated with GP extract can reduce fat load as quantified by Oil Red O staining of intracellular lipid droplets.

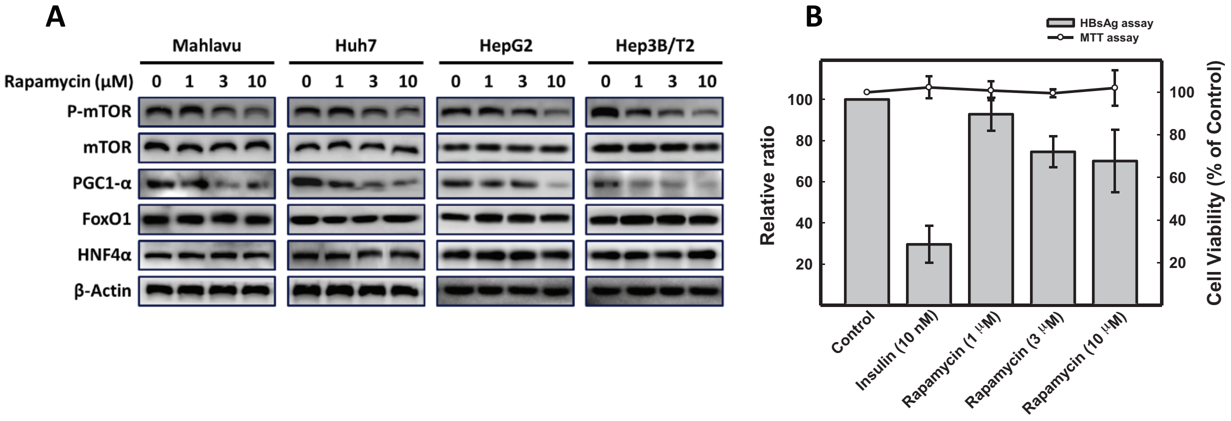

Supplementary Figure S4: Effect of rapamycin in PGC-1α-regulated-HBV gene expression. **(A)** HepG2, Huh7, Hep3B/T2, and Mahlavu cells were treated with rapamycin for 24 h, and then were subjected to Western blot analysis. **(B)** Hep3B/T2 cells were treated with different concentrations of rapamycin in serum-free DMEM medium for 48 h. HBsAg was determined by ELISA. Insulin was used as a positive control.
